# Association of dietary live microbe intake with kidney stone disease in US adults: a real-world cross-sectional study

**DOI:** 10.3389/fnut.2024.1463352

**Published:** 2024-10-21

**Authors:** Zhongyi Zheng, Xiaoming Cao

**Affiliations:** Department of Urology, The First Hospital of Shanxi Medical University, School of Medicine, Shanxi Medical University, Taiyuan, China

**Keywords:** dietary live microbes, kidney stone disease, food-gut-health axis, NHANES, cross-sectional study

## Abstract

**Background:**

Kidney stone disease (KSD) is a common urological condition linked with hypertension, chronic kidney disease, and other health issues. Although the gut microbiome has a notable association with KSD formation, the relationship between dietary live microbes and KSD risk remains underexplored.

**Methods:**

This study utilized data from the NHANES surveys conducted between 2007 and 2016 to analyze the association between dietary live microbe intake and KSD. Dietary intake data were obtained through 24-h dietary recall interviews conducted by trained professionals. Participants were categorized into three groups based on Sanders’ classification system of dietary live microbe intake: low, medium, and high. The intake levels were determined by estimating the live microbe content in foods. Weighted logistic regression analysis was employed to account for the complex survey design and to assess the impact of different levels of live microbe intake on KSD risk.

**Results:**

A total of 20,380 participants were included in the study. Participants with low, medium, and high dietary microbe intake represented 33, 39, and 28% of the cohort, respectively. The adjusted odds ratios (ORs) for KSD were 0.78 (95% CI, 0.65–0.93) in the high dietary live microbe group compared to the low group (*p* < 0.05). Subgroup analyses revealed no significant interactions between dietary live microbe intake and gender, age, BMI, hypertension, or diabetes status.

**Conclusion:**

Higher dietary live microbe intake group may be associated with a reduced risk of KSD. Further prospective studies are necessary to validate these findings and to elucidate the specific mechanisms and optimal intake levels of dietary microbes.

## 1 Introduction

Kidney stone disease (KSD) is one of the most common urological disorders, characterized by the deposition of inorganic substances (such as crystalline salts) and organic components (such as urinary macromolecules) in the renal parenchyma or pelvic system, leading to stone formation (1). Globally, the prevalence of KSD is continuously increasing (2). Epidemiological studies indicate that over 10% of the U.S. population is affected by kidney stones, with a high recurrence rate exceeding 50% (3, 4). Individuals with kidney stones are also at increased risk for hypertension (5), chronic kidney disease (6), and renal cancer (7). The economic burden associated with KSD has also escalated, with costs in the U.S. rising from an estimated $2 billion in 2000 to over $10 billion in 2006 (1). These challenges underscore the urgent need for effective prevention strategies.

Given that KSD formation is commonly associated with diet, metabolic disorders, genetic factors, environmental influences, and underlying medical conditions, it is increasingly recognized as a chronic metabolic disease (8). The human microbiome, which encompasses all microorganisms living in and on the human host, is a key area of study, particularly the gut microbiome. Functionally, it interacts with host cells to perform various biological processes and regulate overall host metabolism (9). The gut microbiome is significantly influenced by diet, and differences in gut microbial communities contribute to the formation of kidney stones (10, 11).

Consuming safe, live microorganisms through daily diet may interact with the mucosal surfaces of the digestive tract, modulate the immune system, enhance gut function, and improve the body’s ability to reduce susceptibility to chronic diseases (12). Marco et al. proposed a classification system using NHANES data to define and estimate dietary intake of live microbes. They suggested assessing existing evidence from dietary databases to quantify the health benefits of consuming live microbes, leveraging data from observational studies like NHANES (12, 13). Based on this classification, literature has explored the associations between dietary live microbes and conditions such as depression (14), cognitive function (15), sarcopenia (16), and cardiovascular diseases (17).

However, no studies have yet evaluated the relationship between dietary live microbe intake and kidney stones. Therefore, this study aims to explore the association between dietary live microbe intake and KSD using NHANES data from 2007 to 2016.

## 2 Materials and methods

### 2.1 Study population

**Figure 1:**
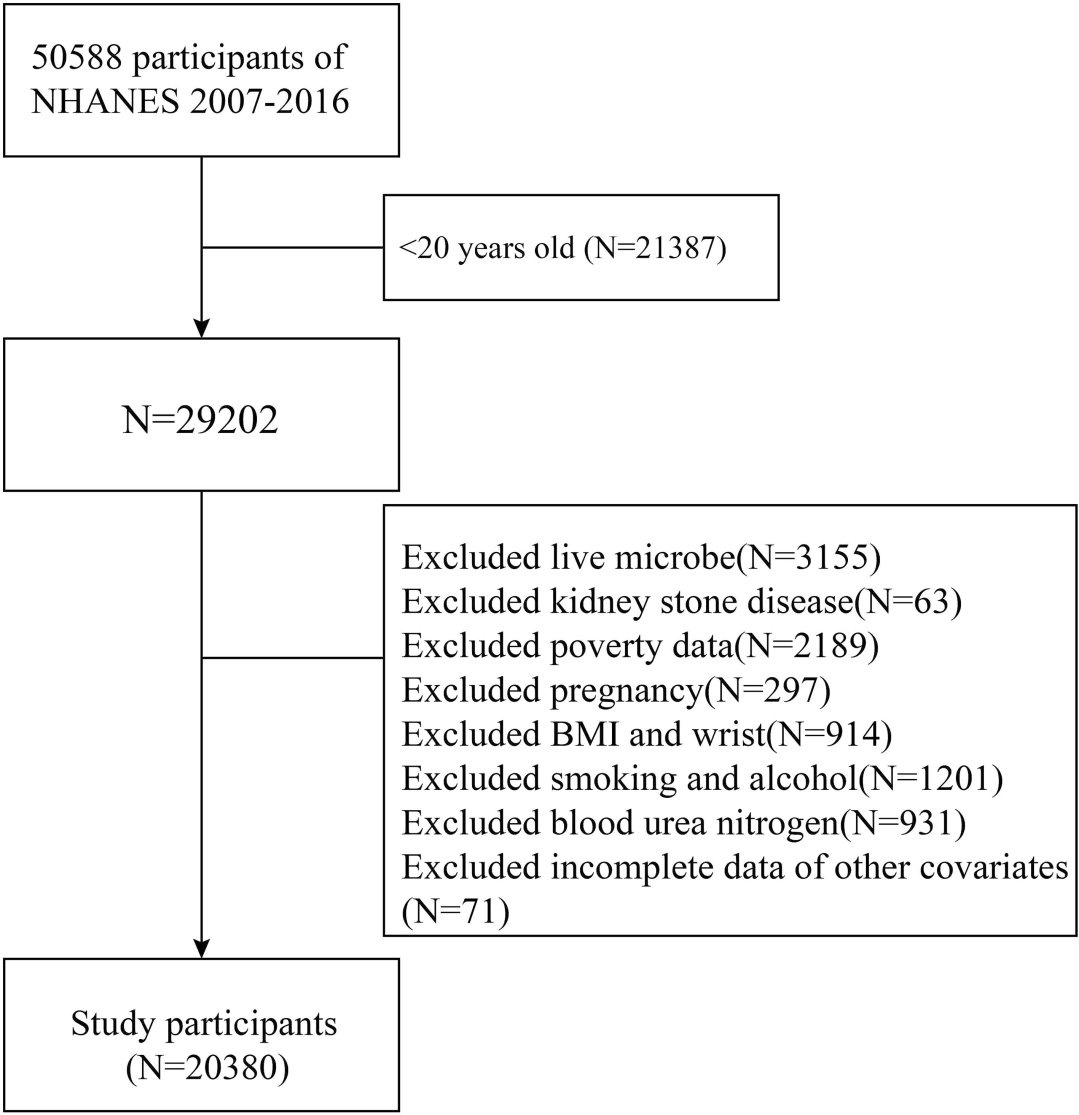
Participant flowchart.

The NHANES database comprises a series of studies designed to evaluate the health and nutritional status of individuals in the United States. To investigate the association between dietary live microbe intake and kidney stone disease (KSD), data from six NHANES cycles (2007–2016) were used to obtain an adequate sample size. Individuals were excluded if they lacked information on dietary live microbe intake or KSD status, were under 20 years old, or had incomplete data on key covariates. These covariates included age, gender, race, poverty income ratio (PIR), marital status, education level, smoking and drinking status, body mass index (BMI), waist circumference, diabetes, hypertension, total energy intake, total water intake, total cholesterol (TC), triglycerides (TG), high-density lipoprotein (HDL), serum creatinine (Scr), blood urea nitrogen (BUN), and uric acid (UA). Ultimately, 20,380 participants were included in the study (Figure 1).

### 2.2 Dietary live microbe data

Dietary intake data were obtained through face-to-face interviews conducted by trained professionals using the 24-h dietary recall method to collect information on all consumed foods and beverages. While this method is subject to recall bias due to participants’ reliance on memory, NHANES employs rigorous quality control procedures to mitigate such bias. Interviewers are trained to assist participants in accurately recalling their dietary intake, using tools such as portion size models, detailed probing questions, and multiple pass methods to reduce underreporting or overreporting (18). Additionally, NHANES uses automated systems to minimize interviewer bias and standardize data collection (19).

The NHANES database includes 9,388 food codes categorized into 48 subgroups. A panel of four experts in the field estimated the live microbe content (per gram) for these food items. Foods were classified into three levels of live microbe content: low (<10^4 CFU/g), medium (10^4–10^7 CFU/g), and high (>10^7 CFU/g). The three participants groups of dietary live microbe were categorized as follows in this study: Low dietary live microbe group (all foods are Lo); Medium dietary live microbe group (any foods are Med but not Hi); High dietary live microbe group (any foods are Hi) (13, 14).

### 2.3 Covariates

The analysis considered the following covariates: sex, age, marital status, education level, race, PIR, diabetes, hypertension, total energy intake, total water intake, BMI, waist circumference, smoking status, and drinking status. Additionally, laboratory measurements provided information on TC, TG, HDL, Scr, BUN, and UA.

### 2.4 Definition of KSD

The primary outcome measure was whether participants had a history of kidney stone disease (KSD). Data related to KSD during the selected cycles were collected in participants’ homes using the Computer-Assisted Personal Interview (CAPI) system, conducted by extensively trained interviewers. Participants were asked, “Have you ever had kidney stones?” If they responded affirmatively, they were classified as having KSD.

### 2.5 Statistical analysis

According to NHANES analysis guidelines, our analysis considered the complex sampling design and sample weights from the mobile examination centers. Data analysis was performed using the “survey” package in R to conduct weighted analyses, which account for the complex survey design of NHANES. Continuous variables were expressed as mean ± standard deviation (SD), and categorical variables were expressed as numbers (percentages). All means and SDs for continuous variables and percentages for categorical variables were weighted. Baseline characteristics between those with and without KSD were compared using t-tests or Mann–Whitney tests for continuous variables and chi-square tests for categorical variables. Weighted univariate analyses were conducted to identify variables that differed significantly between subjects with and without KSD. Additionally, weighted multivariable regression analyses were performed to evaluate the association between dietary live microbe group and KSD, calculating odds ratios (OR) and 95% confidence intervals (CI) to describe these associations. To avoid bias due to the deletion of samples with missing covariates, we conducted multiple imputations as a sensitivity analysis. Stratified analyses and interaction tests were conducted for specific variables, including sex, age (<40 or ≥ 40 years), diabetes status (no or yes), hypertension status (no or yes), and BMI, to assess potential modifiers of the relationship between dietary live microbe group and KSD.

All analyses were conducted using R Statistical Software (The R Foundation).^1^ A *p*-value <0.05 was considered statistically significant.

## 3 Results

### 3.1 Baseline characteristics of the included participants

Table 1 presents the weighted demographic and medical characteristics of participants stratified by the presence of kidney stone disease (KSD). Overall, the study included 20,380 participants with a weighted mean age of 47.4 years. The overall prevalence of KSD was 9.7%. The prevalence of KSD in the low, medium, and high dietary live microbe groups was 11.0, 9.7, and 8.6%, respectively. Significant differences (*p* < 0.05) were observed between individuals with and without KSD in terms of age, BMI, waist circumference, sex, race, marital status, drinking status, smoking status, hypertension, diabetes, dietary live microbe intake group, BUN, UA, Scr, TC, TG, and HDL levels. Participants with KSD exhibited higher values in the following characteristics: age (46.7 ± 16.7 vs. 53.2 ± 15.6 years, *p* < 0.05), BMI (28.75 ± 6.62 vs. 30.47 ± 7.00 kg/m^2^, *p* < 0.05), and waist circumference (98.61 ± 16.19 vs. 104.57 ± 16.67 cm, *p* < 0.05). They also had higher rates of diabetes (30% vs. 45%, *p* < 0.05) and hypertension (8.3% vs. 18%, *p* < 0.05), as well as elevated levels of BUN, UA, Scr, TC, TG, and lower levels of HDL (*p* < 0.05 for all comparisons).

**Table 1:** Basic characteristics of participants by kidney stones disease among US adults.

		KSD		
Characteristic	Total (*N* = 20,380)	No (*N* = 18,403)	Yes (*N* = 1977)	*p* Value
Age (years)	47.4 ± 16.7	46.7 ± 16.7	53.2 ± 15.6	<0.001
BMI(Kg/m2)	28.91 ± 6.68	28.75 ± 6.62	30.47 ± 7.00	<0.001
Waist, cm	99.19 ± 16.33	98.61 ± 16.19	104.57 ± 16.67	<0.001
Gender, n (%)				<0.001
Female	10,146 (51%)	9,297 (51%)	849 (43%)	
Male	10,234 (49%)	9,106 (49%)	1,128 (57%)	
Race, n (%)				<0.001
Mexican American	3,075 (15.1%)	2,813 (15.3%)	262 (13.3%)	
Non-Hispanic Black	4,025 (19.7%)	3,786 (20.6%)	239 (12.1%)	
Non-Hispanic White	9,244 (45.4%)	8,111 (44.1%)	1,133 (57.3%)	
Other Hispanic	2,074 (10.2%)	1,854 (10.1%)	220 (11.1%)	
Other Race	1,962 (9.6%)	1,839 (9.8%)	123 (6.2%)	
Education, n (%)				0.6
High school or equivalent	4,657 (22%)	4,214 (22%)	443 (23%)	
Less than high school	4,768 (15%)	4,270 (15%)	498 (16%)	
More than high school	10,955 (63%)	9,919 (63%)	1,036 (61%)	
PIR (%)	3.01 ± 1.66	3.01 ± 1.67	3.00 ± 1.61	0.8
Marital Status, n (%)				<0.001
Having a partner	12,212 (63%)	10,930 (62%)	1,282 (70%)	
No partner	4,454 (18%)	3,934 (18%)	520 (21%)	
Unmarried	3,714 (19%)	3,539 (20%)	175 (9.0%)	
Alcohol Status, n (%)				<0.001
Former drinking	3,596 (15%)	3,114 (14%)	482 (20%)	
Heavy drinking	4,139 (21%)	3,833 (22%)	306 (16%)	
Mild to moderate drinking	9,860 (53%)	8,925 (53%)	935 (54%)	
Never drinking	2,785 (11%)	2,531 (11%)	254 (11%)	
Smoking Status, n (%)				<0.001
Never Smoking	11,112 (55%)	10,167 (55%)	945 (48%)	
Smoking	9,268 (45%)	8,236 (45%)	1,032 (52%)	
Hypertension, n (%)	7,333 (32%)	6,340 (30%)	993 (45%)	<0.001
Diabetes, n (%)	2,538 (9.3%)	2,099 (8.3%)	439 (18%)	<0.001
Group, n (%)				0.027
Low dietary live microbe group	7,540 (33%)	6,772 (33%)	768 (37%)	
Medium dietary live microbe group	8,164 (39%)	7,375 (39%)	789 (39%)	
High dietary live microbe group	4,676 (28%)	4,256 (28%)	420 (25%)	
Total KCAL(Kcal)	2,161.45 ± 966.88	2,167.27 ± 972.64	2,107.39 ± 910.12	0.2
Total water(g)	3,096.70 ± 1,543.85	3,105.97 ± 1,553.61	3,010.62 ± 1,447.67	0.063
BUN(mg/dL)	13.46 ± 5.21	13.35 ± 5.10	14.53 ± 6.02	<0.001
Scr(mg/dL)	0.89 ± 0.32	0.88 ± 0.31	0.94 ± 0.38	<0.001
UA(mg/dL)	5.46 ± 1.39	5.44 ± 1.38	5.65 ± 1.48	<0.001
TG(mg/dL)	157.73 ± 133.49	155.70 ± 128.60	176.58 ± 171.24	<0.001
TC(mg/dL)	195.54 ± 41.90	195.81 ± 41.80	193.02 ± 42.76	0.013
HDL(mg/dL)	53.24 ± 16.72	53.63 ± 16.82	49.70 ± 15.34	<0.001

PIR, poverty income ratio; BMI, body mass index; TC, total cholesterol; TG, triglycerides; HDL, high-density lipoprotein; Scr, serum creatinine; BUN, blood urea nitrogen; UA, uric acid.

### 3.2 Univariate analysis

Table 2 lists the results of the univariate analysis. The study found that the risk of kidney stone disease (KSD) increased with higher age, BMI, waist circumference, BUN, Scr, and UA levels (*p* < 0.05). Additionally, the presence of hypertension and diabetes was associated with an increased risk of KSD (*p* < 0.05). Compared to females, males had a higher risk of developing KSD (*p* < 0.05). Non-Hispanic White individuals had an increased risk of KSD compared to Mexican American individuals, while Non-Hispanic Black individuals had a decreased risk (*p* < 0.05). Moreover, unmarried individuals had a lower risk of KSD compared to those with partners (*p* < 0.05). Smoking was also associated with an increased risk of KSD compared to non-smoking participants (*p* < 0.05). Notably, individuals in the high dietary live microbe intake group had a lower risk of KSD compared to those in the low intake group (OR = 0.78, 95% CI 0.65–0.93, *p* < 0.05).

**Table 2:** Univariate analysis of variables with kidney stones disease.

Variable	OR (95% CI)	*p*-value
Gender		
Female	1.00 (Reference)	
male	1.37 (1.17–1.60)	<0.001
Age	1.02 (1.02–1.03)	<0.001
Race		
Mexican American	1.00 (Reference)	
Non-Hispanic Black	0.63 (0.50–0.78)	<0.001
Non-Hispanic White	1.52 (1.29–1.80)	<0.001
Other Hispanic	1.22 (0.94–1.59)	0.14
Other Race	0.91 (0.65–1.27)	0.57
Education		
High school or equivalent	1.00 (Reference)	
Less than high school	1.03 (0.86–1.23)	0.72
More than high school	0.95 (0.81–1.12)	0.58
Family PIR	1.0 (0.95–1.04)	0.80
Marital status		
Having a partner	1.00 (Reference)	
No partner	1.04 (0.88–1.24)	0.61
Unmarried	0.40 (0.31–0.52)	<0.001
Smoking status		
Never Smoking	1.00 (Reference)	
Smoking	1.34 (1.17–1.54)	<0.001
Alcohol status		
Never drinking	1.00 (Reference)	
Former drinking	1.45 (1.10–1.90)	0.009
Heavy drinking	0.70 (0.51–0.96)	0.026
Mild to moderate drinking	1.00 (0.77–1.31)	>0.99
BMI	1.04 (1.03–1.04)	<0.001
Waist	1.02 (1.02–1.02)	<0.001
Total energy	1.00 (1.00–1.00)	0.10
Total water	1.00 (1.00–1.00)	0.042
Group		
Low dietary live microbe group	1.00 (Reference)	
High dietary live microbe group	0.78 (0.65–0.93)	0.007
Medium dietary live microbe group	0.89 (0.76–1.04)	0.14
Hypertension		
No	1.00 (Reference)	
Yes	1.89 (1.66–2.16)	<0.001
Diabetes		
No	1.00 (Reference)	
Yes	2.50 (2.17–2.89)	<0.001
BUN	1.04 (1.03–1.05)	<0.001
Scr	1.44 (1.23–1.68)	<0.001
UA	1.11 (1.05–1.17)	<0.001
TG	1.00 (1.00–1.00)	<0.001
TC	1.00 (1.00–1.00)	0.017
HDL	0.98 (0.98–0.99)	<0.001

PIR, poverty income ratio; BMI, body mass index; TC, total cholesterol; TG, triglycerides; HDL, high-density lipoprotein; Scr, serum creatinine; BUN, blood urea nitrogen; UA, uric acid; OR, odds ratio; CI, confidence interval.

### 3.3 Association between dietary live microbe intake with KSD

The results of the multivariable regression analysis are presented in Table 3. Overall, higher dietary live microbe intake group was associated with a lower incidence of kidney stone disease (KSD) across all models (*p* < 0.05). Compared to the low dietary live microbe intake group, the adjusted odds ratio (OR) for KSD in the high intake group was 0.78 (95% CI, 0.65–0.93) in the unadjusted model (*p* < 0.05). In Model 1, adjusted for age and sex, the adjusted OR for KSD in the high dietary live microbe group was 0.77 (95% CI, 0.64–0.93) compared to the low intake group (*p* < 0.05). In subsequent models (Models 2 to 4), further adjustments were made for race, education, family poverty income ratio, marital status, smoking status, alcohol status, BMI, waist circumference, diabetes, hypertension, total energy intake, total water intake, BUN, UA, Scr, TC, TG, and HDL. The association between higher dietary live microbe intake and reduced KSD risk remained statistically significant in these models (all trends *p* < 0.05). In Model 4, after adjusting for the aforementioned covariates, the adjusted OR for KSD in the high dietary live microbe group was 0.79 (95% CI, 0.64–0.98) compared to the low intake group (*p* < 0.05).

**Table 3:** Association between dietary live microbe intake group and kidney stones disease.

Model	Low dietary live microbe	Medium dietary live microbe OR (95% CI)	*p-*value	High dietary live microbe OR (95% CI)	*p-*value
Crude	1.00 (Reference)	0.89 (0.76–1.04)	0.14	0.78 (0.65–0.93)	0.007
Model 1	1.00 (Reference)	0.84 (0.71–0.99)	0.033	0.77 (0.64–0.93)	0.007
Model 2	1.00 (Reference)	0.82 (0.69–0.97)	0.022	0.73 (0.60–0.89)	0.003
Model 3	1.00 (Reference)	0.84 (0.71–1.00)	0.052	0.77 (0.63–0.94)	0.012
Model 4	1.00 (Reference)	0.86 (0.72–1.03)	0.1	0.79 (0.64–0.98)	0.032

Crude was model with no adjustment for covariates. Model 1 was adjusted for age and gender. Model 2 was adjusted for Model 1, and race, education, family poverty income ratio, marital status. Model 3 was adjusted for Model 2, and smoking Status, alcohol Status, bmi, waist, diabetes and hypertension. Model 4 was adjusted for Model 3, and total energy, total water, total cholesterol, blood urea nitrogen, triglyceride, uric acid, high-density lipoprotein cholesterol and serum creatinine. OR, odds ratio; CI, confidence interval.

Based on Sanders’ classification, unpasteurized fermented foods and probiotic supplementation fall under high dietary live microbe intake. This finding is consistent with recent studies proving that probiotic supplementation, such as with *Oxalobacter formigenes*, Lactobacillus, Bifidobacterium, and *Bacillus subtilis*, can reduce intestinal oxalate absorption, which may help prevent kidney stone formation (20, 21). Additionally, a cross-sectional study showed that a higher intake of fermented vinegar was statistically significantly associated with a lower risk of kidney stone formation (22).

### 3.4 Subgroup analyses

**Figure 2:**
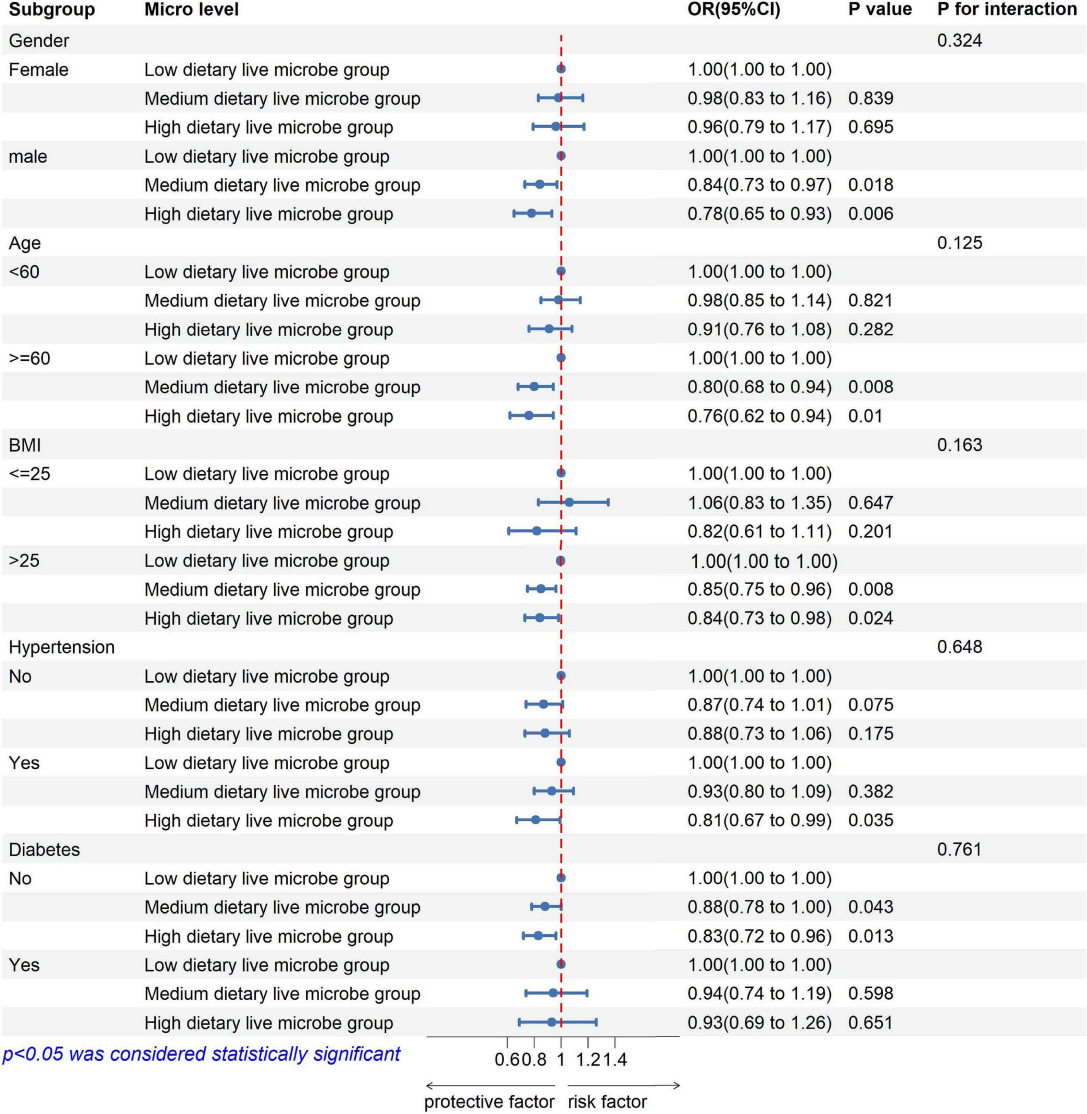
Subgroup analysis of the association between high dietary live microbe group and prevalence of kidney stones disease.

Subgroup analyses were conducted based on sex, age, BMI, hypertension, and diabetes status (Figure 2; Supplementary Table 2). The results indicated no significant interactions between dietary live microbe intake and these stratified variables regarding the risk of kidney stone disease (KSD; P for interaction >0.05). Specifically, male participants, those aged ≥60 years, and those with a BMI >25 exhibited a reduced risk of KSD in the medium and high dietary live microbe intake groups. In the hypertensive subgroup, the high dietary live microbe group had an OR (95% CI) of 0.81 (0.67–0.99) for KSD. Among non-diabetic participants, the ORs (95% CI) for KSD were 0.88 (0.78–1.00) for the medium dietary live microbe group and 0.83 (0.72–0.96) for the high dietary live microbe group, compared to the low intake group. However, the protective effect of high dietary live microbe intake on KSD was attenuated in the presence of diabetes (all *p* < 0.05).

### 3.5 Sensitivity analysis

We conducted multiple imputations to reduce sample bias caused by excluding covariates with missing data. The final results showed that, in the fully adjusted Model 4, the odds ratio (OR) for KSD in the high dietary live microbe group was 0.82 (95% CI, 0.68–0.99, *p* < 0.05) compared to the low intake group. The correlation results remained stable (Supplementary Table 3).

## 4 Discussion

Our study utilized a nationally representative sample of US adults and observed a decrease in the prevalence of kidney stone disease (KSD) among individuals with higher dietary live microbe intake group. The results of the multivariable regression analysis indicated that higher dietary live microbe intake group is associated with a lower risk of KSD. Furthermore, subgroup analyses suggested that the presence of diabetes might influence the relationship between dietary live microbe intake group and KSD. Specifically, our study found that the protective effect of higher dietary live microbe intake group on KSD was attenuated in individuals with diabetes. These findings suggest that higher dietary live microbe intake may be beneficial in preventing KSD.

The association between KSD and the gut microbiome has been well established (10, 21). A meta-analysis of eight studies indicated that patients with KSD have characteristic gut dysbiosis, with significant differences in the overall abundance of microbial communities between KSD patients and controls (23). Urinary oxalate is a key risk factor for KSD, and the gut plays a crucial role in oxalate balance and subsequent oxalate homeostasis (9, 24, 25). Current microbial research on KSD treatment has largely focused on the gut microbiota capable of degrading oxalate (26–30). The gut-kidney axis in KSD is not limited to oxalate-degrading gut microbiota (e.g., *Oxalobacter formigenes*) (31). In KSD patients, the functional activity of gut microbiota involved in oxalate degradation, lipid, carbohydrate, and energy metabolism, glycan synthesis, and amino acid biosynthesis is altered. These findings emphasize the complex interactions between the gut microbiome and KSD, suggesting that interventions targeting the gut microbiome could be a promising strategy for KSD prevention and treatment (32–34).

A metagenome-wide association study (MWAS) has shown that inter-individual differences in gut microbiome composition are influenced by various factors, including lifestyle, diet, disease, and medication, with diet being the most critical (35). The stone-promoting effects of high salt and high oxalate intake, and the preventive effects of increased fruit, vegetable, juice, and water intake, are mediated, at least in part, by changes in gut microbiome composition and metabolic function (36). According to Sanders’ classification, foods that have been pasteurized or processed at high temperatures are considered low in live microbes. Fresh, unpeeled vegetables and fruits are classified as medium, while unpasteurized fermented foods and probiotic supplements are classified as high (13). Increasing the intake of live microbes may be a crucial strategy for improving KSD outcomes. Several studies adopting Sanders’ classification method have shown that diets rich in live microbes are associated with various positive health outcomes, including healthier metabolism, lower BMI (37), reduced cardiovascular disease risk (17), lower depression risk (14), and better cognitive function (15). Probiotic interventions, such as those with *Oxalobacter formigenes*, Lactobacillus paragasseri UBLG-36, and other Lactobacillus strains, have been shown to alter the gut microbiome, affecting the activity of oxalate-degrading bacteria and ultimately reducing oxalate levels and stone formation (26, 28, 38). In our study, high dietary live microbe intake was associated with a lower risk of KSD, while medium intake did not show a significant association in all analyses. The differences in microbial strains and inter-individual variations in the gut microbiome may explain these health outcome discrepancies. It is also essential to consider that, besides live microbes, other dietary components may contribute to health-related parameters. In our study, total energy and total water intake were included in the analysis to strengthen our results. Further evidence, especially in different disease states, is needed to inform dietary recommendations for live microbes. Additionally, Microbiome-targeted interventions, such as probiotic supplementation or dietary modifications aimed at enhancing beneficial microbial populations, represent promising strategies for kidney stone prevention. As research in this area advances, randomized controlled trials and mechanistic studies are needed to validate the efficacy of these interventions and identify the most effective microbial strains and dosages for reducing kidney stone risk.

The potential link between high dietary live microbe intake group and reduced KSD risk can be attributed to several factors. First, probiotics can reduce urinary oxalate levels by influencing the degradation, absorption, and transport of oxalate in the gut, thus maintaining oxalate homeostasis and reducing the risk of KSD (30, 39, 40). Second, safe live microbes can promote the production of metabolites such as short-chain fatty acids (SCFAs), which can downregulate the expression of SLC26A3, a transporter responsible for oxalate absorption in the ileum, cecum, and colon. This results in lower urinary oxalate levels and reduced renal calcium oxalate (CaOx) crystal deposition in rats, contributing to the prevention of kidney stones (41, 42). Overall, these mechanisms highlight the complex interactions between the gut microbiome and KSD. Further mechanistic studies are needed to elucidate the therapeutic advantages of dietary microbes in the prevention and management of KSD.

Our subgroup analysis revealed interesting differences in the association between dietary live microbe intake group and KSD risk. Notably, the protective effect of high dietary live microbe intake was attenuated in individuals with diabetes. The increased risk of KSD in type 2 diabetes patients is primarily attributed to insulin resistance, which is associated with altered renal ammonium secretion, increased urinary acidification, hypocitraturia, and hypercalciuria, all of which contribute to the formation of uric acid and calcium stones (43). The gut microbiome plays a crucial role in the development and progression of both KSD and diabetes (10, 44). Additionally, the use of medications for type 2 diabetes has been linked to changes in the gut microbiome (45, 46), which may partially influence the impact of live microbes on KSD.

NHANES’s rigorous quality control procedures and complex sampling design enabled us to evaluate the association between dietary live microbe intake and KSD in a large, nationally representative sample of U.S. adults. However, several limitations must be acknowledged. First, it is important to recognize the limitation of our study’s cross-sectional design. This design only allows us to examine associations at a single point in time, making it impossible to establish causality. While we observed significant associations between higher dietary live microbe intake and a lower risk of KSD, the temporal sequence of events cannot be determined, and we cannot infer causal relationships. Future longitudinal studies or randomized controlled trials are needed to confirm the causal relationships between dietary live microbe intake and KSD risk. Second, the method used for dietary live microbe classification was based on expert consensus, which may be less accurate than direct measurement methods. While this approach provides a useful framework for categorizing live microbe intake, the lack of microbiome data, particularly those related to dietary microbes, limits our ability to comprehensively understand how these microbes influence disease states. Third, our study population consisted of U.S. adults, limiting the generalizability of the findings to other populations. Another limitation of our study is the reliance on self-reported dietary data, which may introduce recall bias. Participants might inaccurately report their dietary intake, leading to potential misclassification of dietary exposure. While NHANES employs multiple methods to reduce recall bias, including trained interviewers and standardized recall tools, the potential for bias cannot be entirely eliminated. Recognizing these limitations, future research should aim to overcome these challenges, possibly through longitudinal designs, larger and more diverse population samples, and more precise microbial measurement techniques to determine the optimal dosage and types of live microbes for preventing kidney stones.

## 5 Conclusion

Our study indicates that high dietary live microbe intake group is associated with a lower risk of KSD. However, given the limitations of our findings, large prospective studies are necessary to further validate the association between dietary live microbe intake and KSD and to elucidate the underlying mechanisms.

## Data Availability

The datasets presented in this study can be found in online repositories. The names of the repository/repositories and accession number (s) can be found in the article/Supplementary material.

## Footnotes

^1^http://www.R-project.org
