# Supplementary material for: Association of dietary live microbe intake with kidney stone disease in US adults: a real-world cross-sectional study
Source: Front Nutr. 2024 Oct 21;11:1463352. doi: 10.3389/fnut.2024.1463352 (PMC11532051; doi:10.3389/fnut.2024.1463352)
Supplement: Supplementary file 1 [file Table_1.DOCX]

Supplementary Table 1 Basic characteristics of participants by dietary group among U.S. adults.

| **Characteristic** | **Overall, (N = 20380 )** | **Dietary Group** | | | **P-value** |
| --- | --- | --- | --- | --- | --- |
|  |  | **Low dietary live microbe group, (N = 7540 )** | **Medium dietary live microbe group, (N = 8164 )** | **High dietary live microbe group, (N = 4676 )** |  |
| **Age (years)** | 47.4±16.7 | 45.6±16.6 | 48.9±16.9 | 47.3±16.4 | <0.001 |
| **BMI(Kg/m2)** | 28.91±6.68 | 29.58±7.04 | 28.74±6.50 | 28.37±6.40 | <0.001 |
| **Waist, cm** | 99.19±16.33 | 97.72±15.74 | 100.92±16.98 | 98.77±16.07 | <0.001 |
| **Gender, n (%)** |  |  |  |  | <0.001 |
| **Female** | 10,146 (51%) | 3,459 (46%) | 4,148 (52%) | 2,539 (54%) |  |
| **male** | 10,234 (49%) | 4,081 (54%) | 4,016 (48%) | 2,137 (46%) |  |
| **Race, n (%)** |  |  |  |  | <0.001 |
| **Mexican American** | 3,075 (15.1%) | 1,043 (13.8%) | 1,466 (17.9%) | 566 (12.1%) |  |
| **Non-Hispanic Black** | 4,025 (19.7%) | 2,042 (27.1%) | 1,401 (17.2%) | 582 (12.4%) |  |
| **Non-Hispanic White** | 9,244 (45.4%) | 3,021 (40.1%) | 3,638 (44.6%) | 2,585 (55.3%) |  |
| **Other Hispanic** | 2,074 (10.2%) | 717 (9.5%) | 873 (10.7%) | 484 (10.4%) |  |
| **Other Race** | 1,962 (9.6%) | 717 (9.5%) | 786 (9.6%) | 459 (9.8%) |  |
| **Education, n (%)** |  |  |  |  | <0.001 |
| **High school or equivalent** | 4,657 (22%) | 1,963 (27%) | 1,816 (21%) | 878 (18%) |  |
| **Less than high school** | 4,768 (15%) | 2,123 (21%) | 1,945 (15%) | 700 (9.3%) |  |
| **More than high school** | 10,955 (63%) | 3,454 (52%) | 4,403 (64%) | 3,098 (73%) |  |
| **Family_PIR** | 3.01±1.66 | 2.59±1.62 | 3.10±1.65 | 3.38±1.61 | <0.001 |
| **Marital_Status, n (%)** |  |  |  |  | <0.001 |
| **Having a partner** | 12,212 (63%) | 4,177 (58%) | 5,063 (64%) | 2,972 (67%) |  |
| **No partner** | 4,454 (18%) | 1,792 (20%) | 1,771 (18%) | 891 (16%) |  |
| **Unmarried** | 3,714 (19%) | 1,571 (22%) | 1,330 (18%) | 813 (17%) |  |
| **Alcohol_Status, n (%)** |  |  |  |  | <0.001 |
| **Former drinking** | 3,596 (15%) | 1,517 (17%) | 1,414 (15%) | 665 (12%) |  |
| **Heavy drinking** | 4,139 (21%) | 1,698 (26%) | 1,555 (19%) | 886 (19%) |  |
| **Mild to moderate drinking** | 9,860 (53%) | 3,245 (46%) | 4,029 (55%) | 2,586 (60%) |  |
| **Never drinking** | 2,785 (11%) | 1,080 (11%) | 1,166 (11%) | 539 (9.1%) |  |
| **Smoking_Status, n (%)** |  |  |  |  | <0.001 |
| **Never Smoking** | 11,112 (55%) | 3,801 (49%) | 4,586 (56%) | 2,725 (59%) |  |
| **Smoking** | 9,268 (45%) | 3,739 (51%) | 3,578 (44%) | 1,951 (41%) |  |
| **Hypertension, n (%)** | 7,333 (32%) | 2,790 (33%) | 3,015 (33%) | 1,528 (29%) | <0.001 |
| **Diabetes, n (%)** | 2,538 (9.3%) | 995 (9.9%) | 1,097 (10%) | 446 (7.4%) | <0.001 |
| **KSD, n (%)** | 1,977 (9.7%) | 768 (11%) | 789 (9.7%) | 420 (8.6%) | 0.027 |
| **Total KCAL(Kcal)** | 2,161.45±966.88 | 2,103.25±1,000.31 | 2,146.38±939.12 | 2,251.06±958.69 | <0.001 |
| **Total water(g)** | 3,096.70±1,543.85 | 2,910.87±1,558.04 | 3,139.54±1,507.40 | 3,256.05±1,555.25 | <0.001 |
| **BUN(mg/dL)** | 13.46±5.21 | 12.87±5.16 | 13.72±5.33 | 13.81±5.02 | <0.001 |
| **Scr(mg/dL)** | 0.89±0.32 | 0.90±0.33 | 0.89±0.34 | 0.87±0.26 | <0.001 |
| **UA(mg/dL)** | 5.46±1.39 | 5.60±1.42 | 5.44±1.39 | 5.33±1.35 | <0.001 |
| **TG(mg/dL)** | 157.73±133.49 | 163.01±128.80 | 156.79±143.93 | 152.80±123.19 | 0.001 |
| **TC(mg/dL)** | 195.54±41.90 | 193.09±41.70 | 195.73±42.08 | 198.15±41.73 | <0.001 |
| **HDL(mg/dL)** | 53.24±16.72 | 50.64±15.58 | 54.02±16.85 | 55.24±17.44 | <0.001 |
| **PIR;poverty income ratio , BMI;body mass index ,TC; total cholesterol , TG;triglycerides , HDL;high-density lipoprotein, Scr;serum creatinine ,BUN; blood urea nitrogen , and UA;uric acid KSD:kidney stones disease.** | | | | | |

Supplementary Table 2 Subgroup analyses of the association between dietary live microbe intake group and kidney stones disease.

| **Subgroup** | **Variable** | **OR(95%CI)** | **P value** | **P for interaction** |
| --- | --- | --- | --- | --- |
| **Gender** |  |  |  | 0.324 |
| **Female** | Low dietary live microbe | 1.00(Reference) |  |  |
|  | Medium dietary live microbe | 0.98(0.83-1.16) | 0.839 |  |
|  | High dietary live microbe | 0.96(0.79-1.17) | 0.695 |  |
| **male** | Low dietary live microbe | 1.00(Reference) |  |  |
|  | Medium dietary live microbe | 0.84(0.73-0.97) | 0.018 |  |
|  | High dietary live microbe | 0.78(0.65-0.93) | 0.006 |  |
| **Age Group** |  |  |  | 0.125 |
| **<60** | Low dietary live microbe | 1.00(Reference) |  |  |
|  | Medium dietary live microbe | 0.98(0.85-1.14) | 0.821 |  |
|  | High dietary live microbe | 0.91(0.76-1.08) | 0.282 |  |
| **>=60** | Low dietary live microbe | 1.00(Reference) |  |  |
|  | Medium dietary live microbe | 0.8(0.68-0.94) | 0.008 |  |
|  | High dietary live microbe | 0.76(0.62-0.94) | 0.01 |  |
| **BMI Group** |  |  |  | 0.163 |
| **<=25** | Low dietary live microbe | 1.00(Reference) |  |  |
|  | Medium dietary live microbe | 1.06(0.83-1.35) | 0.647 |  |
|  | High dietary live microbe | 0.82(0.61-1.11) | 0.201 |  |
| **>25** | Low dietary live microbe | 1.00(Reference) |  |  |
|  | Medium dietary live microbe | 0.85(0.75-0.96) | 0.008 |  |
|  | High dietary live microbe | 0.84(0.73-0.98) | 0.024 |  |
| **Hypertension** |  |  |  | 0.648 |
| **No** | Low dietary live microbe | 1.00(Reference) |  |  |
|  | Medium dietary live microbe | 0.87(0.74-1.01) | 0.075 |  |
|  | High dietary live microbe | 0.88(0.73-1.06) | 0.175 |  |
| **Yes** | Low dietary live microbe | 1.00(Reference) |  |  |
|  | Medium dietary live microbe | 0.93(0.8-1.09) | 0.382 |  |
|  | High dietary live microbe | 0.81(0.67-0.99) | 0.035 |  |
| **Diabetes** |  |  |  | 0.761 |
| **No** | Low dietary live microbe | 1.00(Reference) |  |  |
|  | Medium dietary live microbe | 0.88(0.78-1.00) | 0.043 |  |
|  | High dietary live microbe | 0.83(0.72-0.96) | 0.013 |  |
| **Yes** | Low dietary live microbe | 1.00(Reference) |  |  |
|  | Medium dietary live microbe | 0.94(0.74-1.19) | 0.598 |  |
|  | High dietary live microbe | 0.93(0.69-1.26) | 0.651 |  |

Supplementary Table 3 The results of Association between Dietary Live Microbes and Kidney stones disease after multiple imputations.

| **model** | **Low Dietary Live Microbe** | **Medium Dietary Live Microbe**  **OR (95% CI)** | **Pvalue** | **High Dietary Live Microbe**  **OR (95% CI)** | **Pvalue** |
| --- | --- | --- | --- | --- | --- |
| **Crude** | 1.00 (Reference) | 0.89 (0.76-1.03) | 0.11 | 0.79 (0.67-0.93) | 0.004 |
| **Model 1** | 1.00 (Reference) | 0.84 (0.72-0.98) | 0.025 | 0.78 (0.66-0.92) | 0.004 |
| **Model 2** | 1.00 (Reference) | 0.83(0.71-0.97) | 0.019 | 0.75 (0.63-0.90) | 0.002 |
| **Model 3** | 1.00 (Reference) | 0.86 (0.73-1.01) | 0.058 | 0.80(0.66-0.96) | 0.017 |
| **Model 4** | 1.00 (Reference) | 0.87 (0.74-1.03) | 0.1 | 0.82 (0.68-0.99) | 0.043 |
| Crude was model with no adjustment for covariates.  Model 1 was adjusted for age and gender.  Model 2 was adjusted for Model 1, and race,education,family poverty income ratio,marital status.  Model 3 was adjusted for Model 2, and smoking Status,alcohol Status, bmi ,waist , diabetes and hypertension.  Model 4 was adjusted for Model 3, and total energy,total water,total cholesterol, blood urea nitrogen,triglyceride, uric acid, high-density lipoprotein cholesterol and serum creatinine.  OR, odds ratio; CI, confidence interval. | | | | | |
